# 

**Published:** 1911-04-01

**Authors:** 


Telegraphic Address: Ospedale, London.

THE MODERN MEDICAL AND INSTITUTIONAL JOURNAL.

Telephone Number: 2734 Gerrard.

NEW SERIES. No. 217, Vol. IX. No. 1289, Vol. L.

SATURDAY, APRIL 1ST, 1911.

PRICE THREEPENCE

